# A 3D roaming and collision detection algorithm applicable for massive spatial data

**DOI:** 10.1371/journal.pone.0229038

**Published:** 2020-02-21

**Authors:** Mingxia Xie, Xinqiang Niu

**Affiliations:** 1 Key Laboratory of Urban Land Resources Monitoring and Simulation, Ministry of Land and Resources, Shenzhen, China; 2 Changjiang Institute of Survey, Planning, Design and Research, Wuhan, China; Central South University, CHINA

## Abstract

In this paper, a novel 3D roaming algorithm considering collision detection and interaction is proposed that adopts a triangle mesh to organize and manage massive spatial data and uses a customized bounding box intersector to rapidly obtain the potential collided triangles. The proposed algorithm can satisfy the requirements of timeliness and practicability during complicated large 3D scene collision detection. Moreover, we designed a method to calculate the collision point coordinates according to the spatial position relation and distance change between the virtual collision detection sphere and triangles, with the triangle edges and three vertices being considered. Compared to the methods that use the native intersector of OpenSceneGraph (OSG) to obtain the collision point coordinates, the calculation efficiency of the proposed method is greatly improved. Usually, when there is a big split/pit in the scene, the viewpoints will fly off the scene due to the fall of the collision detection sphere, or the region interior cannot be accessed when the entrance of some local region (e.g., internal grotto) of the scene is too small. These problems are solved in this paper through 3D scene-path training and by self-adaptively adjusting the radius of the virtual collision detection sphere. The proposed 3D roaming and collision detection method applicable for massive spatial data overcomes the limitation that the existing roaming and collision detection methods are only applicable to 3D scenes with a small amount of data and simple models. It provides technical supports for freewill browsing and roaming of indoor/outdoor and overground/underground of the 3D scene in cases of massive spatial data.

## Introduction

With the rapid development of 3D spatial data acquisition technologies (e.g., 3D laser scanner, oblique photography, UAV measurement, and BIM) as well as spatial object modelling technologies, the availability of 3D spatial data with various scales or different degrees of details has been greatly improved. The data size and complexity of 3D scenes have sharply increased while critically improving the verisimilitude of 3D scenes. Presently, 3D spatial data acquisition and modelling technologies have been widely applied to digital twins and the product life-cycle management of engineering (i.e., water resource and hydropower engineering, constructional engineering, underground pipe network engineering, and railroad and bridge engineering). During the application process, the constructed 3D scene is both a 3D geospatial environment model with a macro-region and a complicated 3D virtual scene with a micro and exquisite building model. When the viewing point enters from the macroscopic scene into the micro indoor scene, if it does not consider the collision detection problem and roams and analyzes indoor, it will lead to the problem of incorrect analysis and calculation due to the inaccurate capture or acquisition of the viewing point. Moreover, during the roaming process, it has the phenomena of penetration (e.g., flying into the ground and passing through the wall) that do not conform with real situations, which makes it difficult to roam and analyze indoors or makes it unable to operate.

Scene roaming and collision detection are the core technologies of virtual scenes. Their effects directly influence the reality of the whole virtual scene [1]. The existing roaming algorithms considering collision detection mainly construct the target bounding box to judge the collision relation among actual objects and find the balance between the close joint degree of bounding box with the actual collision object surface with the collision detection effectiveness; thus, roaming in 3D scenes is realized [2][3]. Existing algorithms have mainly been applied to 3D scenes with less data volume and to simple models. There are still no clear and effective solutions regarding how to realize high-efficient roaming in consideration of real-time collision detection and interaction in massive and complicated 3D scenes so as to effectively avoid the phenomenon of penetration that does not conform to actual situations. Besides, the problem of inaccurate capturing of the viewing point remains. However, it has critical significance in the fields of digital twins and engineering product life-cycle management for integrated management, analysis, roaming, and applications of massive indoor/outdoor and overground/underground spatial data. In addition to improving the sense of reality for virtual scenes, it is convenient for users’ indoor operations, such as searching and analyzing.

## Current situation analysis

In a 3D scene, people usually observe an object with their eyes. If the viewing point moves, the surrounding scenery moves reversely relative to the viewing point. This process leads to a scene roaming effect in the brain. Collision is caused by user interaction and object movement during roaming [4]. During scene roaming, when the system receives the user input, it needs to judge if the new viewing points corresponding to the user input will collide with or enter objects in the scene [5] [6].

### Analysis of collision detection methods

There are two types of collision detection methods: intersector detection methods and bounding box detection methods. In intersector detection methods, the iterator is bonded with an intersector to realize the intersection process of the whole scene. The common types of intersectors include straight line intersectors and polygon intersectors. In bounding box detection methods, bounding boxes with simple geometrical features are used to approximately describe complex geometric objects, and then a tree hierarchy is constructed to gradually approach the objects’ geometric model until almost all the geometrical features of the objects are obtained [7–13]. Common types of bounding boxes include axis-aligned bounding box (AABB), oriented bounding box (OBB), discrete orientation polytopes (K-Dop), fixed directions hulls (FDH), and Sphere.

### Analysis of roaming algorithms

The present roaming algorithms are mainly based on 3D engines, such as OpenSceneGraph (OSG) [14] [15] [16], Unity3D [17] [18] [19] [20], and object-oriented graphics rendering engine (OGRE) [21] [22] [23]. They can be applied to 3D scenes with a small amount of data and simple models. OSG is a 3D engine for graphics development that is high-performing, open-source, and cross-platform, which has functions such as large-scale scene paging, dynamic trimming, particle system and shadow, multithreading and multi-display rendering as well as supporting multiple file formats. Therefore, it has good performance in terms of massive data loading and display.

Because the default manipulator of OSG does not consider realizing collision detection, a manipulator needs to be designed that can detect the collision of objects in real-time, calculate the related collision response, and update the display results to avoid the phenomenon of having penetration that does not conform to the real situation. For the existing manipulators being applied to 3D scenes with massive data designed by using an OSG intersector, there are mainly the problems of low efficiency of collision judgement and the collision point coordinates calculation.

Unity3D is a comprehensive game engine, and it includes multiple modules, such as the graphics rendering module, the physical module, the sound module, and the network module. OGRE is a scene-oriented real-time 3D graph engine without network, physical, sound, or topography modules; instead, it is only concentrated on 3D rendering. OGRE implements collision detection and rigid body physics while Unity3D has a built-in PhysX physical engine. OGRE and Unity3D can realize rapid and convenient roaming and collision detection, but their flexibility is low. For example, if a function cannot satisfy the requirement, then it is difficult to be fixed. Moreover, when the data volume is large, scene loading and construction is very inefficient for 3D engines based on Unity3D and OGRE, and the program can even become stuck, which results in a poor roaming experience.

In summary, most of the existing roaming algorithms are only suitable for closed 3D scenes with small data volume and simple models. However, for actual engineering applications, such as water resources and hydropower and underground pipe networks, the constructed 3D virtual scene has a large amount of data and many special-shaped structures as well as a variety of complicated conditions, such as split/pit and interior grottos. The existing roaming algorithms cannot satisfy the demand for complicated 3D macro scene of massive spatial data. In this paper, by using the PageLOD technology and the construction methods of a triangular mesh and intersector in the OSG 3D graphic engine, the bounding box and intersector are applied to collision detection in massive and complicated 3D scenes, a manipulator is designed in consideration of collision detection and intersection function, and thus the roaming requirements of complicated 3D scenes with massive spatial data are satisfied.

## Methods

**Fig 1:**
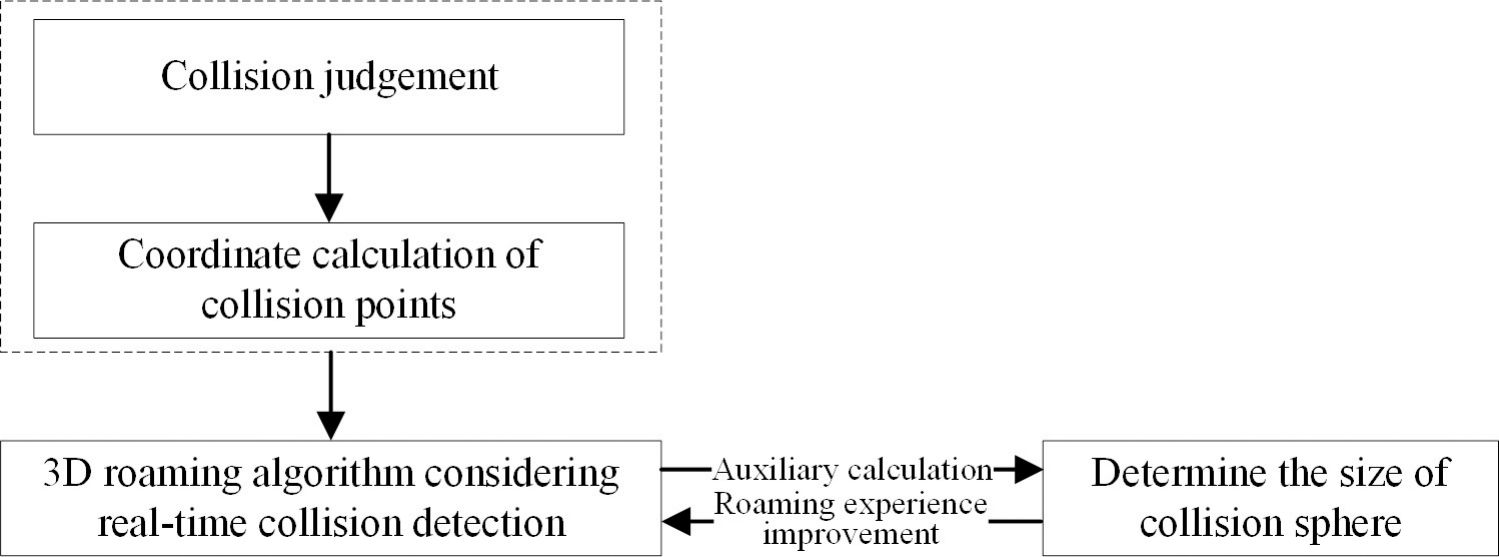
Correlation of each key technology.

The 3D roaming algorithm that supports massive spatial data solves four problems: 1) how to rapidly and correctly detect if a collision occurs in 3D scenes; 2) how to accurately calculate the coordinates of collision points in real time; 3) how to design the 3D roaming algorithm considering real-time collision detection; 4) how to determine the size of the virtual collision sphere. Among them, the collision estimation and the coordinate calculation of the collision points are the basis of solving the others. By using PageLOD technology and the construction methods of a triangular mash and intersector in the OSG 3D graphic engine, a 3D roaming algorithm that supports massive spatial data and considers real-time collision detection can be designed. Through the designed 3D roaming algorithm, the radius size of the collision detection sphere is adjusted. Thus, the roaming experience of massive and complicated 3D scenes is improved (as shown in Fig 1).

The collision time calculation methods, the coordinate calculation method of the collision points, the 3D roaming methods considering collision detection, and the adjustment methods of collision detection sphere radius for massive and complicated 3D scenes are introduced below.

### Collision time estimation based on spatial position relation and distance

The collision time of the occurrence is calculated according to the spatial position relation and distance change between the detection sphere and triangle. If the virtual collision detection sphere is a unit sphere, that is, the radius is 1, then the position of the detection sphere at time t is as follows:

$$C(t)=P_{0}+t\times v,t\in [0,1]$$ (1)

where *P*_0_ is the initial position of the virtual collision detection sphere, *v* is the velocity of movement of the virtual collision detection sphere, and *C*(*t*) is the position of the virtual collision detection sphere at time *t*.

According to the point-to-plane distance formula:

$$D(p)=N∙p+C_{p}$$ (2)

where p is the position coordinates of the point, N is the normal vector of the triangle plane, C_p_ is the plane constant, and *D*(*p*) is the distance from *p* to the triangle plane.

**Fig 2:**
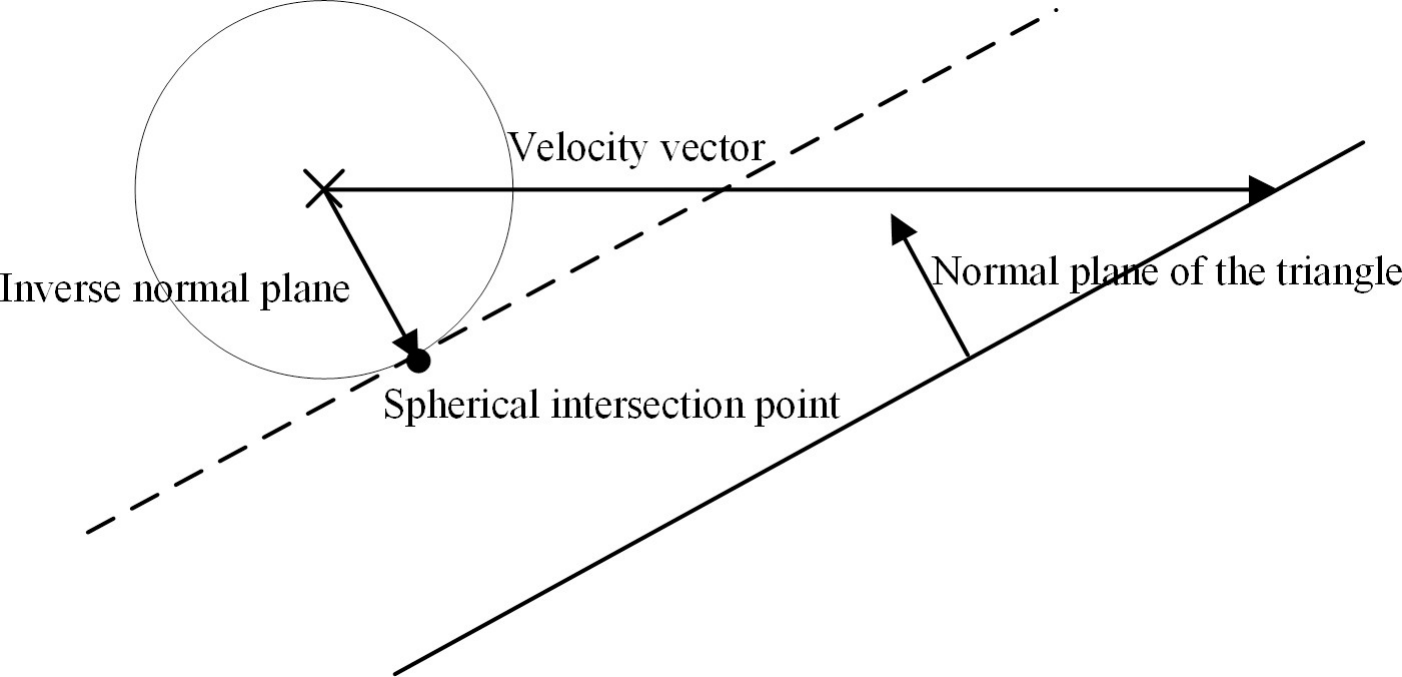
Normal plane of the triangle, inverse normal plane, and velocity vector.

If at some time *t*_0_ the virtual collision detection sphere collides with the triangle plane in the scene, that is, the separation relation is transferred to tangency relation, then the distance from the center of the virtual collision detection sphere to the triangle plane is 1. When the virtual collision detection sphere is about to penetrate the triangle plane, that is, at time t_1_ when the triangle plane transfers from intersection relation to tangency relation, then the distance from the center of the virtual collision detection to the triangle plane is -1. Therefore, at time t∈[t_0,t_1 ], the virtual collision detection sphere and the triangle plane always stay at a state of the intersection. Therefore, the following can be obtained:

$$D(C(t_{0}))=1,D(C(t_{1}))=‐1$$ (3)

where *D*(*C*(*t*_0_)) is the distance from the bounding sphere center to triangle plane at time *t*_0_ and *D*(*C*(*t*_1_)) is the distance from the sphere center to triangle plane at time *t*_1_. By substituting Eq (1) and Eq (2), the following can be obtained:

$$t_{0}=\frac{1‐D(p_{0})}{N∙v},t_{1}=\frac{‐1‐D(p_{0})}{N∙v}$$ (4)

where *D*(*p*_0_) is the distance of the initial position *P*_0_ of the virtual collision detection sphere to the triangle plane; N is the normal vector of the triangle plane; *P*_0_ is the initial position of the virtual collision detection sphere; and *v* is the velocity of the virtual collision detection sphere, as shown in Fig 2.

### Calculation of the collision point coordinates

#### Collision with the triangle plane

**Fig 3:**
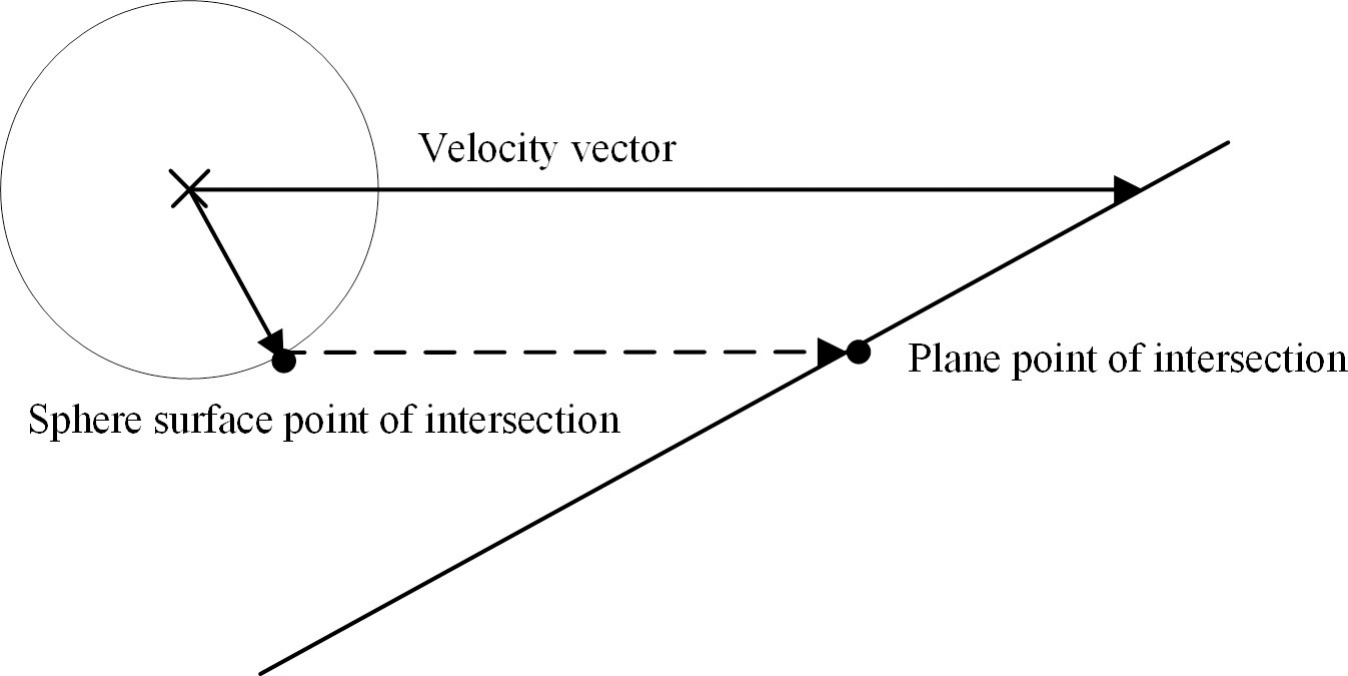
Point of the intersection when the virtual collision detection sphere collides with the triangle plane.

According to the collision detection methods above, when the virtual collision detection sphere collides with the plane of a triangle that constitutes the 3D scene, the collision time t_0_ is calculated. By substituting it into Eq (3), the coordinates of calculation collision point are obtained as follows:

$$p_{intersection}=p_{1}+f_{0}e_{1}\;p_{intersection}=p_{0}‐N+t_{0}\times v$$ (5)

where *p*_intersection_ are the coordinates of the collision point when the virtual collision detection sphere collides with the triangle plane; *P*_0_ is the initial position of the virtual collision detection sphere; *v* is the velocity of the virtual collision detection sphere; N is the normal vector of the triangle plane; and *t*_0_ is the time when the virtual collision detection collides with the plane of a triangle that constitutes the 3D scene, as shown in Fig 3.

#### Collision with the triangle edges and vertices

If the virtual collision detection sphere does not collide with the triangle plane, then further estimation should be conducted for the triangle’s three edges *e*_1_,*e*_2_,*e*_3_ and three vertices *p*_1_,*p*_2_,*p*_3_. If the virtual collision detection sphere collides with triangle edges or vertices (e.g., consider vertex *p*_1_ and edge *e*_1_ = *p*_1_−*p*_2_), then the distance from the center of the sphere to vertex *p*_1_ and edge *e*_1_ is 1. By resolving the distance equation, the collision point coordinates are obtained as follows:

$$p_{intersection}=p_{1}+f_{0}e_{1}$$ (6)

where $f_{0}=\frac{(e_{1}∙v)t‐e_{1}e_{p}}{{\left\|e_{1}\right\|}^{2}}$, e_p_ = p_1_-p_0_, and *e*_*p*_ is the edge connecting point *p*_0_ and point *p*_1_; *v* is the velocity of the virtual collision detection sphere; t is the time point when the virtual collision detection sphere collides with the triangle edge or vertex; and *p*_intersection_ is the collision point coordinates when the virtual collision detection sphere collides with the triangle edge or vertex.

Graphical descriptions of the triangle normal plane, inverse normal plane, and velocity vector during the collision detection and calculation of collision point coordinates are shown in Fig 2 and Fig 3 In addition, the sphere point of the intersection and the plane point of the intersection when the virtual collision detection sphere moves along a direction and collides with a triangle plane that constitutes the 3D scene are shown.

### Design of the 3D roaming algorithm considering real-time collision detection

**Fig 4:**
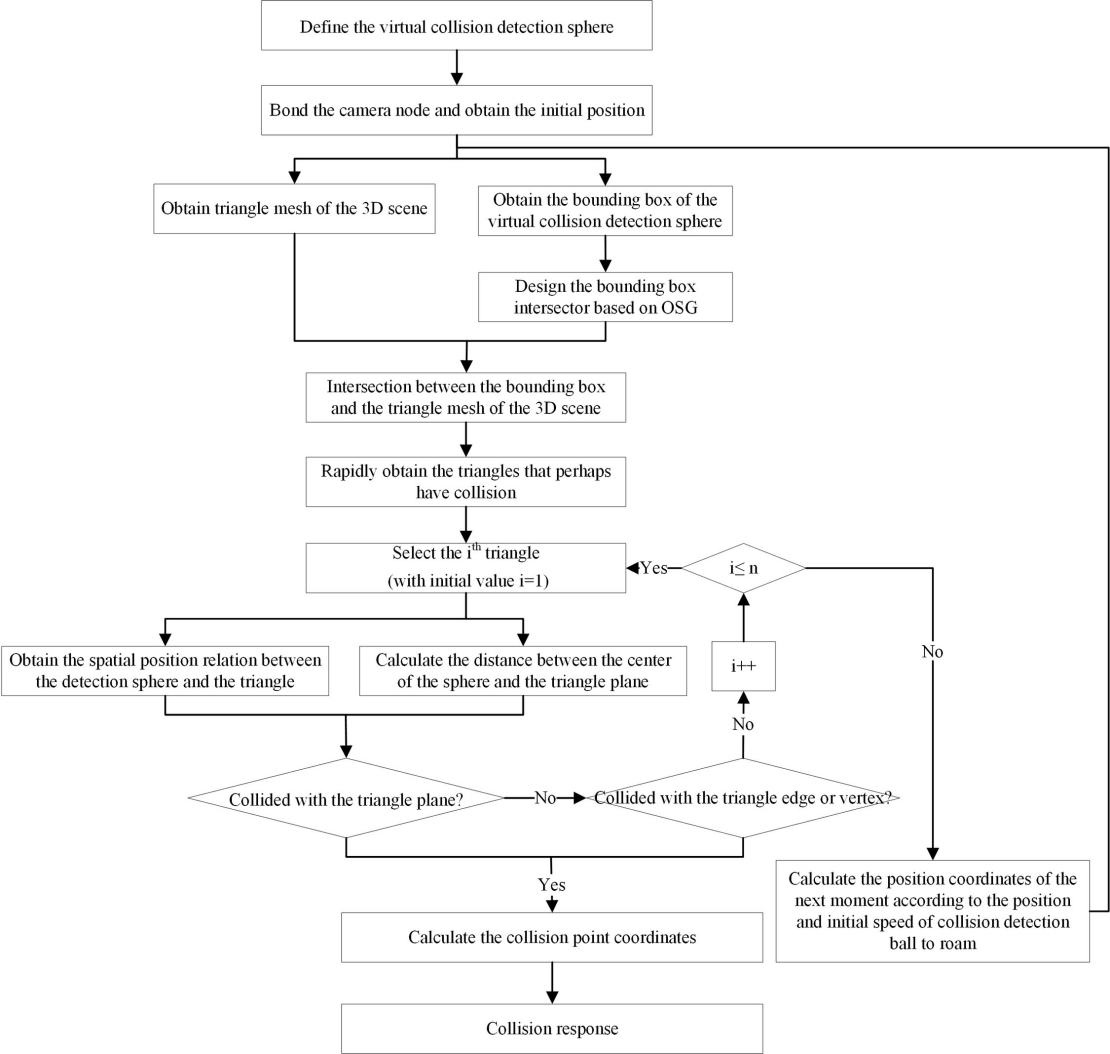
3D roaming and collision detection that supports massive spatial data.

Based on the collision detection estimation method mentioned above, the coordinate calculation method of the collision points, the PageLOD technology, and the target bounding box acquisition and intersection calculation methods of the OSG 3D engine, a 3D roaming algorithm that supports massive spatial data and considers real-time collision detection is designed. In this algorithm, getBoundingBox() is the function, defined in OSG, that obtains its bounding box according to the object types. In addition, the osgUtil::Intersector()function, defined in OSG, judges if the two objects intersect. The specific implementation steps of the 3D roaming algorithm considering real-time collision detection are given below (as shown in Fig 4):

Step1: Define the virtual collision detection sphere, including the sphere radius and initial velocity;
Step2: Bound the camera node to the virtual collision detection sphere and obtain the initial position according to camera viewpoint information;
Step3: Obtain the triangular mesh data of the 3D scenes;
Step4: Use getBoundingBox() in OSG to obtain the bounding box of the virtual collision detection sphere;
Step5: Design bounding box intersector based on osgUtil::Intersector() in OSG and rapidly obtain the triangular mesh that may collide with virtual collision detection sphere in real-time;
Step6: If a collision occurs, obtain the collision time and collision point coordinates, and perform the collision response according to the above-mentioned collision time estimation and collision point coordinate calculation methods;
Step7: If no collision occurs, calculate the position coordinate at the next time point for roaming according to the collision detection sphere position as well as the initial velocity.

When designing the intersector based on osgUtil::Intersector(), it will estimate and screen the collision triangles according to the position relation between the vertex coordinates of various triangles in the mesh and bounding box.

**Fig 5:**
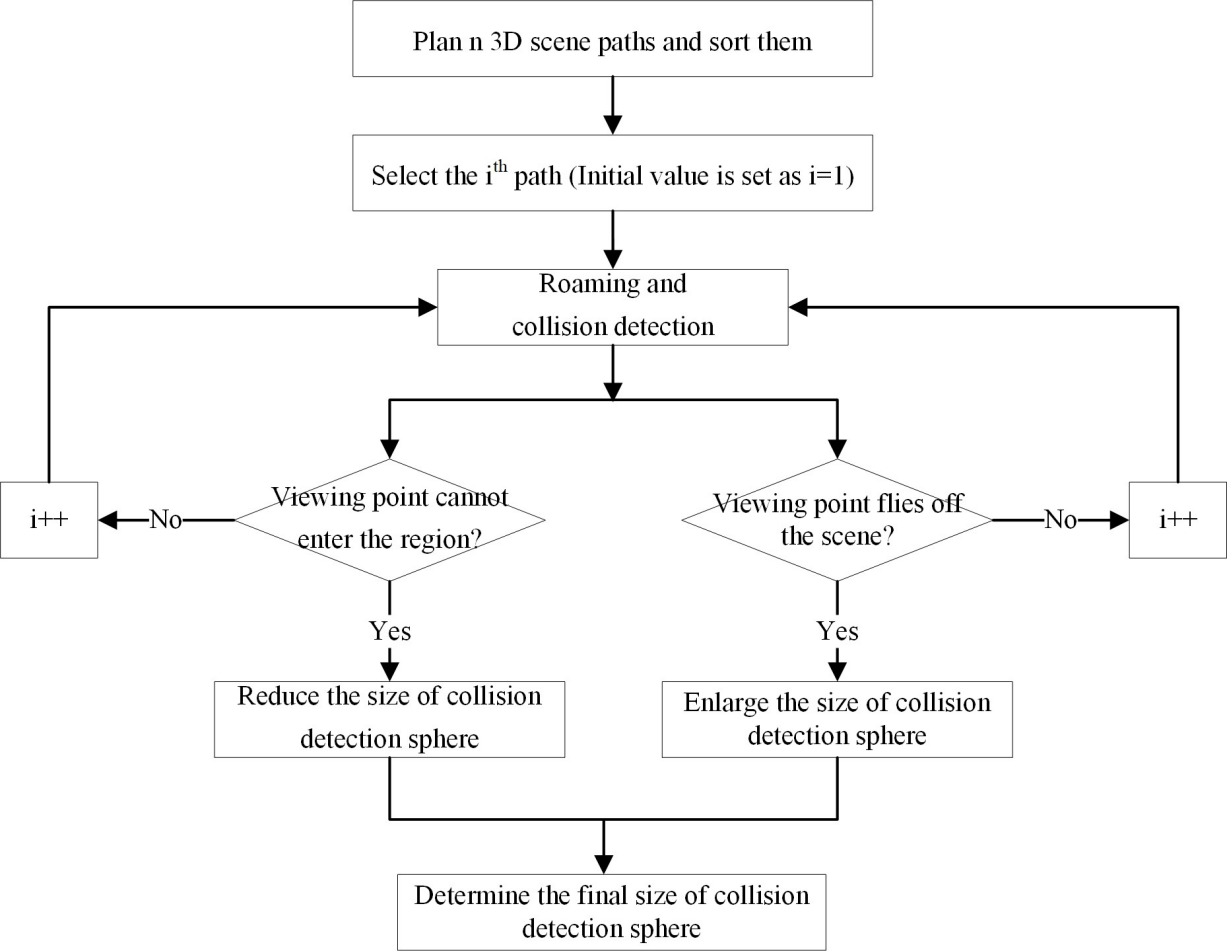
Preliminary estimation function of the relation in intersector.

As shown in Fig 5, where v1, v2, and v3 are the three vertices of the triangle, respectively, and _boundingBox is the bounding box of the virtual detection sphere. When the function returns “true”, it indicates the triangle does not intersect with the bounding box. In this way, the triangles without any possibility of collision can be excluded quickly. During calculation of the collision time and the collision point coordinates, it should be estimated if the collision detection sphere collides with the triangle mesh plane. Additionally, when the detection sphere does not collide with the triangle mesh interior, then the three edges and three vertices of triangle mesh should be further checked one by one.

### Self-adaptive adjustment of the collision detection sphere radius

In this paper, a self-adaptive adjustment method of collision detection sphere radius is designed. This method solves the problem that occurs during the actual roaming and collision detection process. In the process, the viewing point flies off the scene when there is a big split/pit in the 3D scene. Also, it solves the problem where the collision detection sphere cannot enter some local region (e.g., the internal grotto) of the 3D scene due to a small entrance. First, *n* paths are planned randomly in the 3D scene and are numbered, in which *n* is a natural number and *n*≥1 and the specific value should be determined according to the complexity of the scene (with different scenes of roaming and collision detection experiments, the initial value is usually set as *n* = 5).

**Fig 6:**
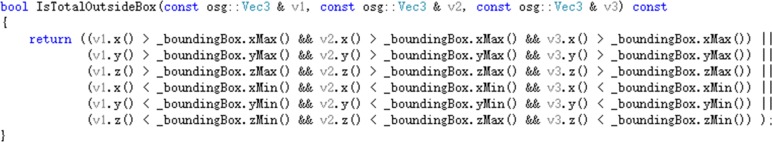
Self-adaptive adjustment process of the virtual collision detection sphere radius.

Afterwards, by using the 3D roaming algorithm considering real-time collision detection introduced in the last section, the first path is selected for roaming. If the viewing point cannot enter into the scene interior during the roaming process, then the radius of the virtual collision detection sphere decreases; if the viewing point flies off the current roaming scene, then the radius of the virtual collision detection sphere increases. Finally, when the training of all planning paths is accomplished, the radius size of the virtual collision detection sphere suitable for actual 3D application scenarios is determined. Moreover, the input collision detection sphere’s radius parameters in the 3D roaming algorithm are adjusted (as shown in Fig 6).

## Experimental analysis

### Experimental data

**Fig 7:**
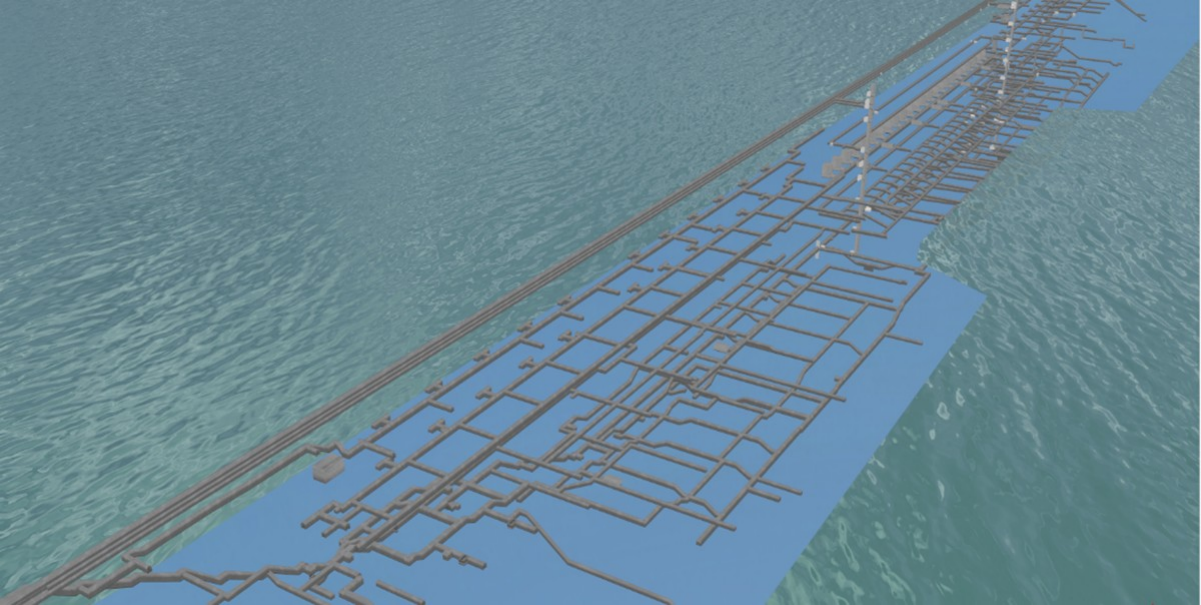
Visualization of the gallery 3D model data.

To verify the effectiveness and operability of the 3D collision roaming and collision detection algorithm that supports massive spatial data, a domestic dam gallery data set was selected for an experiment. Part of the galleries had grottos where monitoring instruments are installed, and the bottoms had drainage grottos and joints. The constructed 3D scene mainly includes basic 3D topographic data, 3D model data of the dam, the monitoring instruments, and the gallery. The total amount of the data was about 3 GB, in which the gallery 3D model data was about 50 MB. Fig 7 shows a visualization of the gallery 3D model data.

### Calculation efficiency comparison of the collision detection and collision point coordinates

**Fig 8:**
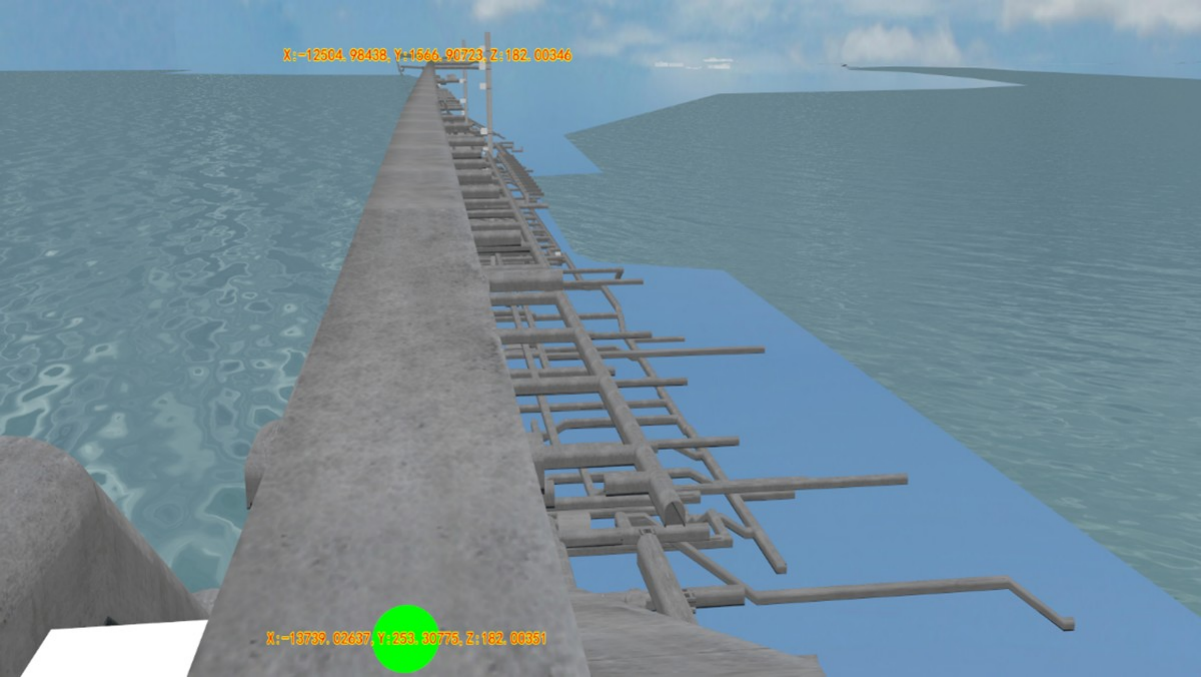
Roaming path 1 (straight-line path without split).

**Fig 9:**
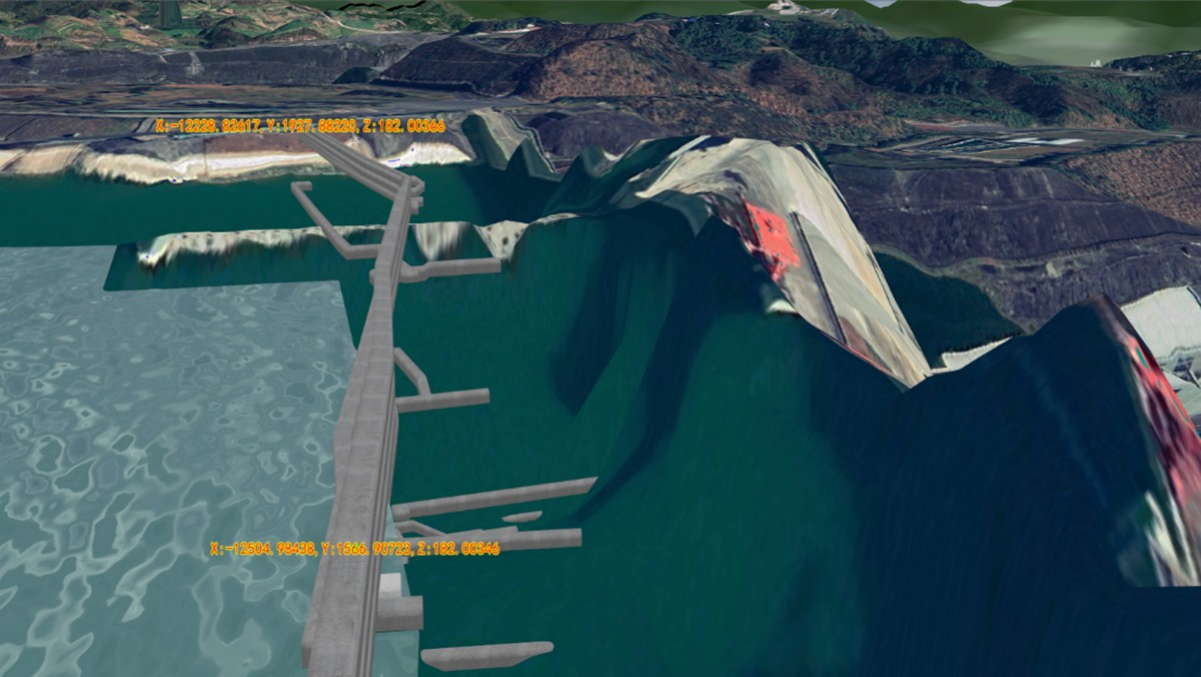
Roaming path 2 (complicated corner path without a split).

**Fig 10:**
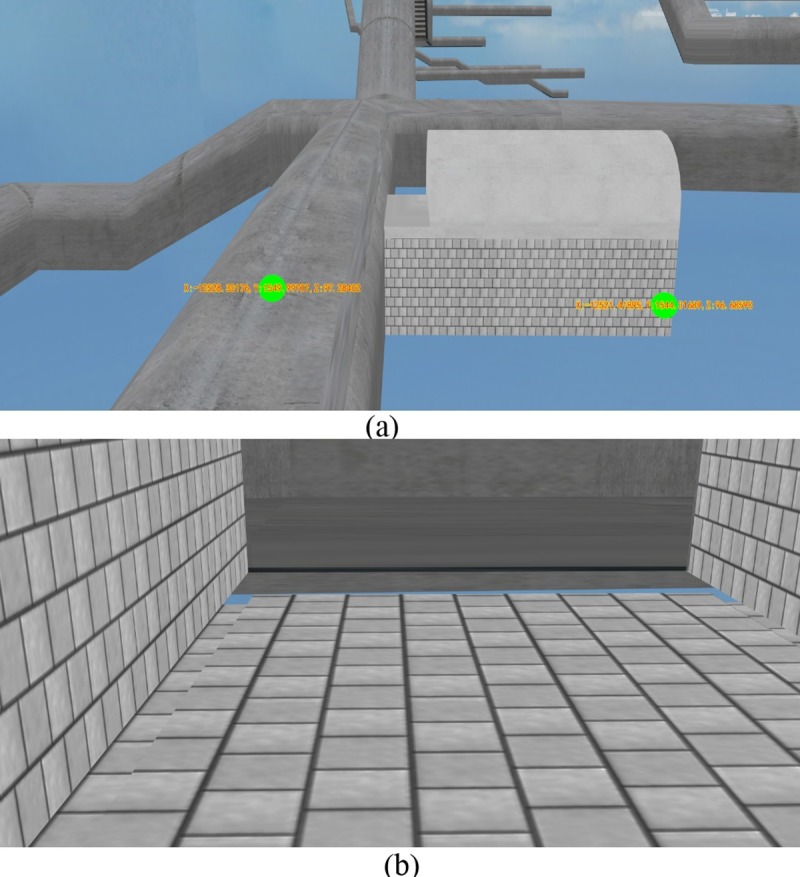
Roaming path 3 (Straight-line path with a split): (a) The starting point and end point of the path; (b) A split in the roaming path.

A comparison was conducted between the method of collision point coordinates calculation proposed in this paper and the polytope intersector osgUtil::PolytopeIntersector based method in OSG. Three roaming paths were first selected. The first one was a straight-line path without a split (as shown in Fig 8, where the coordinates are the starting point and end point of the selected roaming path in the gallery); the second one was a corner path without a split (as shown in Fig 9, where the coordinates are the starting point and end point of the selected roaming path in the gallery); and the third one was a straight-line path with a split (as shown in Fig 10, where the coordinates are the starting point and end point of the selected roaming path in the gallery). Then, the starting points and end points of the paths were obtained, and the initial velocity vector was set for the collision detection sphere, including the magnitude and direction. Finally, the roaming time and completeness of the roaming for each path were compared, and the results are shown in Table 1. The calculation process based on the polytope intersector of OSG is as follows: First, the bounding box of the collision detection sphere is obtained. Then, the function Polytope::setToBoundingBo is used in OSG to transform the bounding box of the collision detection sphere into a polygon. Finally, the function PolytopeIntersector::getIntersections() is used in the intersector to obtain the intersection content, that is, the collision point coordinates.

**Table 1:** A calculation efficiency comparison of the collision detection and collision point coordinates.

roaming path	Coordinates of the starting point(m)	Coordinates of the end point(m)	Initial velocity		roaming time(s)	
			Mangnitude(km/s)	direction	Based on OSG	This method
**Path 1 (Straight-line path without split)**	(-13739, 253, 182)	(-12504, 1566, 182)	0.05	(1, 1, 0)	52.7	38.4
**Path 2 (Corner path without split)**	(-12504, 1566, 182)	(-12228, 1927, 182)	0.05	(1, 1, 0)	roaming not finished, stuck at the corner.	19.03
**Path 3 (Straight-line path with split)**	(-12528, 1549, 97)	(-12521, 1544, 96)	0.05	(1, 1, 0)	roaming not finished, flying off the range of viewing at the split	0.21

Note: The starting and end point coordinates are the defined projection plane coordinates in the system.

The roaming is smooth and without any jam phenomenon in the specific collision response process using the method of this paper ((Table 1), which can satisfy practical demands. By contrast, using the method based on polygon intersector of OSG, the roaming sometimes pauses due to long calculation time or even becomes stuck somewhere in the scene, and thus it cannot satisfy the practical demands.

### Effects comparison analysis of 3D roaming and collision detection

**Fig 11:**
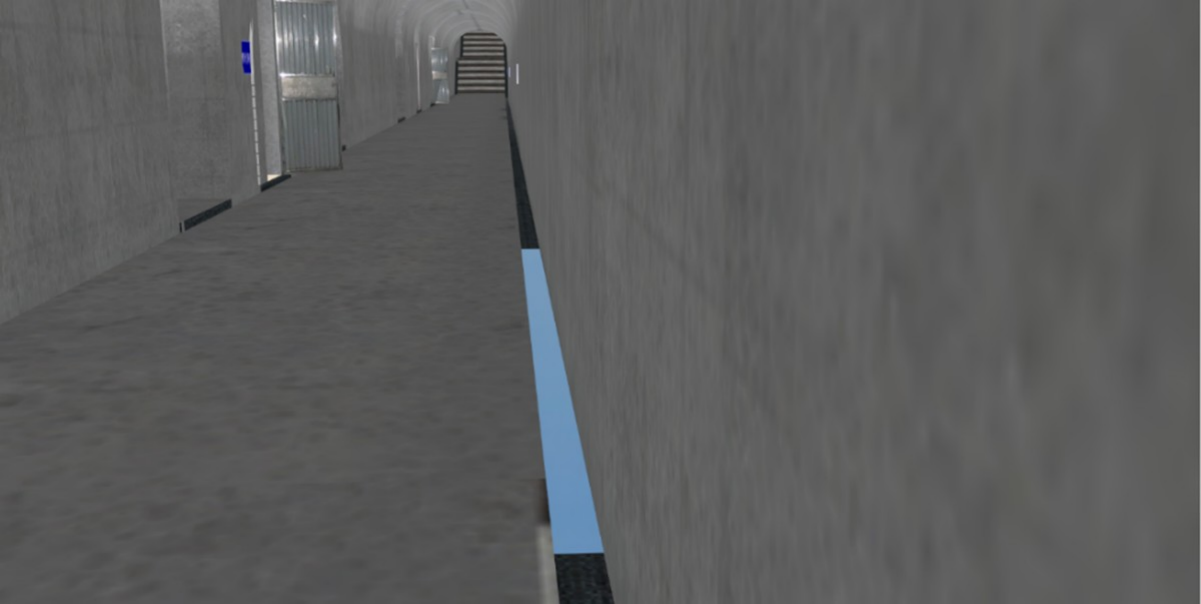
Normal roaming when there is a split in the bottom of the gallery.

**Fig 12:**
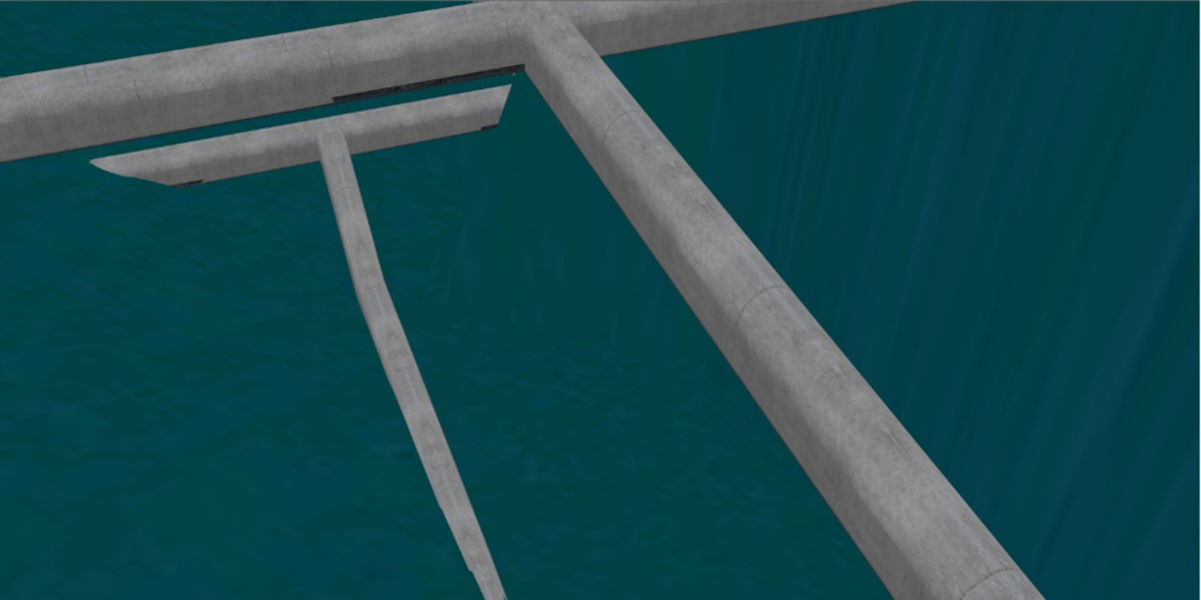
The viewing point flies off the current indoor scene and falls to the ground when there is split in the bottom of the gallery.

**Fig 13:**
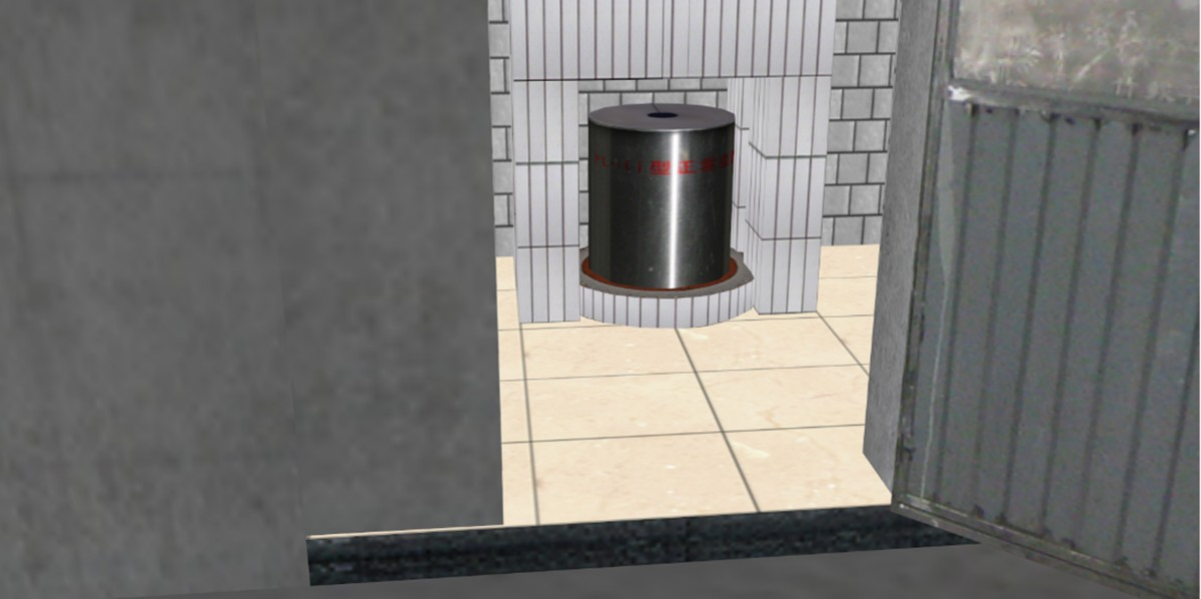
A grotto (or room with small entrance) exists inside the scene.

The roaming performance under the same 3D scene was compared for four different methods to better illustrate the advantages of the 3D roaming and collision detection algorithm proposed in this paper. The proposed method was compared with Unity3D, OGRE, and OSG + Bullet. The comparison results are shown in Table 2. The roaming algorithm proposed in this paper could avoid the condition where the collision detection sphere falls into the split when there is a split in the bottom of the gallery, which resulted in the viewing point flying off the current roaming scene, as shown in Fig 11. In contrast, the viewing points all flew off the current roaming scene when using the other three methods (as shown in Fig 12). In addition, during the roaming process in the gallery, the roaming based on Unity3D and OGRE had continuous jam phenomena and long waiting times during the data loading. As shown in Fig 13, when there is a grotto (or room with small entrance) in the scene, for the roaming algorithms based on Unity3D and OGRE, the sphere sometimes failed to enter it. However, for the proposed method, this situation could be avoided well by self-adaptively adjusting the detection sphere radius.

**Table 2:** Roaming performance comparison of four methods: Method of this paper, Unity3D, OGRE, and OSG + Bullet.

Method	User’s roaming experience in the complicated 3D scene under massive spatial data	Whether flies off current roaming scene	Whether fit for the 3D scene of massive spatial data	Whether fit for complicated 3D model	Whether the virtual collision detection sphere can make a self-adaptive adjustment
**Based on Unity3D**	Jammed and easy to lead to system breakdown	Yes	No	No	No
**Based on OGRE**	Jammed and easy to lead to system breakdown	Yes	No	No	No
**Based on OSG+Bullet**	roaming is smooth	Yes	Yes	No	No
**The method in this paper**	roaming is smooth, and the system runs stably	No	Yes	Yes	Yes

As shown in the table above, given the 3D virtual scenes constructed for large-scale water resources and hydropower engineering, the data volume is large and it usually has a large number of special-shaped structures, as well as complicated conditions like a joint pot and interior grotto. Compared with the existing roaming methods, the 3D scene roaming algorithm designed in this paper has the advantages of fast collision detection, wide applicability, and a good roaming experience for users.

1. By using the open-source features of OSG itself, supporting massive spatial data, and combining the principles observed by collision detection, it reconstructs and designs the technologies, such as the scene triangular mesh and intersector construction. It can satisfy the demands of effectiveness and practicability of collision detection in 3D-scene roaming under the condition of massive spatial data. Moreover, it overcomes the defect of the existing roaming methods being mainly applied to the 3D scenes with less data volume and simple models. Therefore, it has improved the applicability of the 3D roaming method.
2. By adopting the method of 3D scene path roaming training, the radius size of the virtual collision detection sphere is self-adaptively adjusted. It aims to make the sphere size satisfy the demand of roaming collision in 3D scenes, without subjective manual adjustment. It also avoids the problem that viewing point flies off the scene and cannot enter into some scene interior, which enhances the user experience and makes it smoother for operations in virtual scenes, such as roaming, selecting, and analyzing.
3. Given that the algorithm itself is based on the OSG 3D graphic engine and supplements collision detection, it does not consider the mutual physical interaction among objects during the collision process. Hence, the algorithm in this paper cannot be applied to physical analysis calculation and mechanical simulation for the collision process among actual objects.

## Conclusions

This paper proposes a 3D roaming algorithm supporting massive spatial data. It is based on the OSG 3D graphic engine, uses its strong management ability of massive spatial data, combines the construction and management method of scene triangular mesh in OSG, and eventually designs a coordinate calculation method of collision points that considers each element of triangular mesh, such as point, line, and plane, during the 3D scene roaming process. It also puts forward an estimation method of the collision relation between the virtual collision detection sphere and triangular mesh. Based on this, the intersector of the triangular facet and the bounding box is constructed. The radius size of the detection sphere is adjusted self-adaptively by planning and selecting the roaming paths. It can solve the problem where the viewing point flies off the scene from split/pit and cannot roam into the grotto interior during the roaming process of complicated 3D scenes with split/pit or grottos.

The algorithm of this paper extends the OSG manipulator; supplements the support of OSG manipulator for collision detection function; overcomes the defect that the existing roaming methods are mainly applied to 3D scenes with less data volume and simple models; provides technical support for realizing operations, such as random browsing, roaming, searching, and analyzing, of indoor/outdoor and overground/underground of 3D scenes under massive spatial data. This makes it better applicable to actual engineering projects. Furthermore, the algorithm does not consider the texture of the collision objects during the collision detection process nor the complicated physical interaction and mechanical analysis during the collision process. In a subsequent study, the calculation efficiency and relation estimation method for the constructed intersector will be further improved and perfected. Moreover, an analysis of the physical texture of the collision and the complicated physical during the collision process will be conducted. This will make the proposed algorithm not only applicable to the virtual roaming of massive complicated 3D scenes but also support mechanical analysis and simulation calculation for massive complicated 3D scenes.

## Supporting information

S1 Data Gallery model. (IVE)
